# Role of primary care pharmacists in the post-hospital discharge care of patients: a scoping review protocol

**DOI:** 10.1186/s40545-022-00473-5

**Published:** 2022-10-29

**Authors:** Faiza Yahya, Hamde Nazar, Muhammad Abdul Hadi

**Affiliations:** 1 Our Health Partnership, First Floor, 1856 Pershore Road, Cotteridge, Birmingham, B30 3AS UK; 2 School of Pharmacy, Newcastle University, King George VI Building, Newcastle-Upon-Tyne, NE1 7RU UK; 3 College of Pharmacy, QU Health, Qatar University, 2713 Doha, Qatar

**Keywords:** Non-dispensing pharmacists, Clinical pharmacists, General practice, Transition of care, Medicines reconciliation, Transfer of care

## Abstract

**Background:**

Evidence has shown that there is a significant problem with medication safety when patients are transferred between settings. The role of community pharmacists and hospital pharmacists in facilitating transition of care has been well-researched. However, with the developing role of pharmacists in general practice as part of a multi-disciplinary team, little is known about their role in improving transition of care when patients move from secondary to primary care. The key objective of this scoping review is to understand the nature and extent of the role of primary care pharmacists for patients recently discharged from secondary care.

**Methods:**

This scoping review will follow the Joanna Briggs Institute (JBI) methodology for scoping review underpinned by the Arksey and O’Malley methodology and reported in accordance with the Preferred Reporting Items for Systematic Reviews Extension for Scoping Reviews (PRISMA-ScR) guidelines. The following electronic databases will be systematically searched: MEDLINE, EMBASE, PubMed, Cochrane Central Register of Controlled Trials (CENTRAL), Web of Science and NICE Evidence. Reference lists of included full texts will be searched for relevant papers, in addition to grey literature which includes websites of relevant professional organisations. Primary studies, published in the English language that involved a primary care pharmacist-led intervention post-hospital discharge will be included. Two independent reviewers will screen studies against eligibility criteria and use a piloted data extraction form to extract data related to the review questions. The data will be presented in tabular form and assessed for key themes to identify gaps and inform future research.

**Discussion:**

This scoping review will map current evidence surrounding the role of primary care pharmacists in the post-hospital discharge care of patients. Findings will inform ongoing research to support safer transfer-of-care post-hospital discharge and identify ways in which collaboration between healthcare professionals can be improved. This review anticipates guiding the inclusion of patient and public involvement (PPI) at the consultation stage to validate and build on the findings.

**Supplementary Information:**

The online version contains supplementary material available at 10.1186/s40545-022-00473-5.

## Background

There is a recognised need for improving medication safety at hospital discharge and ensuring safer transfer of information about medicines between care transitions [1]. A recent systematic review by Alqenae et al. [2] identified that around 50% of adult patients experience medication errors or unintentional medication discrepancies following hospital discharge. On average 4.4 drug changes are made per patient at hospital discharge, with 76% of patients having three or more drug changes during their hospital stay [3]. Hence, the efficient and timely communication across sectors is crucial. Significant risk of medication-related problems, especially in high-risk patients, has been highlighted in the 7- to 10-day period following hospital discharge and research on the feasibility of timely pharmacist-intervention pathways has been explored to improve continuity of care [4].

Medication errors have a huge burden on the National Health Service (NHS) in England with over 237 million errors made annually across medication processes [5]. One study found that medication-related harm, affecting 1 in 3 older adults, incurs a cost of around £396 m annually to the NHS, of which £243 m could potentially be avoidable. It was shown that 90% of these costs are attributable to hospital re-admissions and 74% related to medication prescribed at hospital discharge [6]. In addition to unnecessary, potentially avoidable costs, reducing medication-related harm on transfer between settings is a priority area for quality improvement identified by the World Health Organization (WHO) as part of the global patient safety challenge ‘Medication without Harm’ [7]. Furthermore, an ageing population and increasing patients with complex polypharmacy [8], studies have shown that patients with multiple medication changes can often be unaware of what the changes are and the reasons for them upon hospital discharge [9]. Qualitative studies have highlighted how patients identified system vulnerabilities and anticipated medication discrepancies upon discharge at a time when they can already be anxious, emotionally and functionally impaired [10]. This communication breakdown can be exceptionally problematic for patients with low health/English literacy or who rely on the support of others for medicines management especially where there are social and functional gaps in post-discharge care [11]. Hence, patient safety in transitions and communications of care for the most vulnerable have been highlighted as research priorities in the United Kingdom [12]. Developments in better digital information systems for safer transfer of information about medicines has been recommended [13] and interventions to improve continuity of care such as the discharge medicines service in community pharmacies have been implemented [14]. Considerable publications have been developed over the years to support better transfer of care [15], with a recent guide published by the Royal College of Physicians (RCP) to support quality improvement projects concerning medication safety at hospital discharge [16].

The role of pharmacists in transitions of care has been well-researched primarily within a hospital setting or community pharmacy. Little has been known about primary care and how pharmacy teams in general practice can improve transition of care between settings. Over recent years there is a recognised growing workload crisis in general practice in the UK [17]. To alleviate some of the workload-related pressures, NHS England (NHSE) in line with the NHS five-year forward view [18] launched the clinical pharmacists in general practice scheme in 2015. Post-implementation, studies have shown that primary care pharmacists (PCPs) have expanded to more patient-facing roles and medicines reconciliation following transfer-of-care forms a significant proportion of their role and integration in general practice [19, 20]. These advancements have recognised the value of PCPs’ expert knowledge and skills in medicines optimisation to manage patients holistically within a multi-disciplinary team [21]. This can be crucial for patients with complex polypharmacy often at a time of vulnerability at post-hospital discharge. Thus the role of pharmacists in medicines optimisation as defined by the National Institute for Health and Care Excellence (NICE) [22] to ensure safe and effective use of medicines can often involve follow-up and liaising with other health care professionals, community pharmacists and or/carers [23].

Although extensive studies have been carried out on the impact of pharmacist interventions in medicines reconciliation [24–26] and medication reviews [27], this has mainly been related to hospital pharmacists and community pharmacists, however, little has been studied specifically on the role of PCPs in general practice in this context. Systematic reviews have been conducted on medication reconciliation by pharmacists in both community and primary care settings [25] and not necessarily differentiating the impact or role of a pharmacist in general practice. Whilst the effectiveness of pharmacist interventions has been researched in these settings, pharmacists have shown to have an impact on resolving drug discrepancies, but further research on clinical relevance of these discrepancies and workload impact has been highlighted [25]. A recent Cochrane review by Redmond et al. [25] primarily evaluating the impact of pharmacist-led interventions in medicines reconciliation for improving transitions of care found that the impact was uncertain due to the reliability of the evidence being low, according to the GRADE approach for Cochrane reviews [28]. For community pharmacists’ role specifically, a systematic review [29] has shown that community pharmacist interventions can improve drug-related problems after discharge, recognising that the role of pharmacists has potential to improve transfer of care. Despite the heterogeneity and variation of studies of an uncontrolled nature included, this systematic review highlighted some valuable lessons to inform the future design and implementation of complex interventions as defined by the Medical Research Council (MRC) [30] and identify cause–effect relations [29]. Furthermore, in a recent systematic review, pharmacist collaborations with physicians have been researched and demonstrated effectiveness at reducing hospital readmissions with recommendations that further rigorous research is required [31]. This review was generalised to pharmacists in any sector, however little is known specifically about how primary care pharmacist colleagues or collaboration between sectors can have an impact on improving continuity of care across transitions. It is known that PCPs integration in general practice is beneficial on patient clinical outcomes and satisfaction [32], as well as positive effects with multi-faceted interventions and interprofessional collaborations [33, 34]. Although reducing GP workload was a main driver for the funding of PCPs, there remains a paucity of evidence on this impact and similarly the impact on quality and safety is important [17]. Whilst a positive reduction in GP workload on emergency admissions is evident, further research is needed on the impact on healthcare systems [35], cost-effectiveness and whether interventions by PCPs are beneficial to transitions of care. Although collaborative medicines optimisation at hospital discharge is promoted [1] and implementation advocated in recent guidance [16], it is still unclear how this is currently practised in primary care.

A preliminary rapid search on MEDLINE, Cochrane and CINAHL Plus has been conducted and no current systematic reviews or scoping reviews on the impact of post-hospital discharge interventions by primary care pharmacists specifically were identified. It is also apparent that there is a lack of controlled trials in primary care concerning improving medicines-related transitions of care. Furthermore, many systematic reviews and meta-analyses to date have been limited with poor and inconsistent descriptions of pharmacist interventions in studies [10]. With the development and varying role of pharmacists in primary care settings, this review will allow an overview relating to the role of PCPs in transfer of care. This scoping review has set out to examine the extent, range, nature of research activity in this topic. Informed by a priori protocol to systematically map the available literature, a scoping review was identified as the most appropriate methodology to review this question, summarise key themes in research to date, and would be more insightful to allow identification of knowledge gaps to inform future research initiatives [36] or determine whether a full systematic review would be valuable [37].

## Methods

This scoping review will be conducted in accordance with the JBI methodology for scoping reviews [38] which follows nine key steps (Additional file 1). This was built upon the original methodology proposed by Arksey and O’Malley [39] and later enhanced [37, 38, 40]. The structure and reporting will be guided by the Preferred Reporting Items for Systematic Reviews Extension for Scoping Reviews (PRISMA-ScR) guidelines [41].

To improve methodological rigour and validity, consultation with key stakeholders (i.e. experts and researchers in the field, consultant pharmacist in primary care and experienced primary care pharmacists) was sought throughout the development of this protocol as recommended by the JBI [38]. The execution and dissemination of the evidence will also share preliminary findings with stakeholders and help inform future implications for practice [37]. The consultation stage will aim to develop a broader understanding of the role of primary care pharmacists and involve community pharmacists, primary care pharmacists, clinicians, and researchers in the field. In addition, dependent on the findings of this review, a patient and public involvement (PPI) consultation will also be considered to add value and identify future research priorities [42].

The Population, Concept and Context (PCC) mnemonic was used to develop the research question, as recommended by the JBI guidelines for scoping reviews. This was used to guide the inclusion and exclusion criteria as well.

### Participants

All patients that are recently discharged from the hospital and have had an intervention by a primary care pharmacist.

### Concept

This review will focus on primary care pharmacist-led interventions among patients recently discharged from the hospital.

### Context

This review will evaluate literature that includes pharmacist-led interventions in a primary care setting, reviewing the location (country) and the types of studies conducted and method design. The review will map which outcome measures have been studied such as medicines reconciliation, medication review, medication counselling, identification of medication errors, reduction of hospital admissions, reduction of GP workload and any financial impacts. Identification of any collaborations of the primary care pharmacists with community pharmacy or physicians will be evaluated.

The specific objectives are:

- To identify literature describing and evaluating role of primary care pharmacists in facilitating transitions of care.
- What type of interventions have been performed by primary care pharmacists on patients recently discharged from hospital?
- What study designs and outcome measures have been used in research evaluating the effectiveness of primary care pharmacist interventions post-hospital discharge?
- What are the nature and opportunities for collaboration between primary care pharmacists and physicians and community pharmacists?

## Eligibility criteria

### Inclusion and exclusion criteria

This review will include articles looking at adult patients (aged 18 or above) recently discharged from hospital and must have had an intervention from a primary care pharmacist in a primary care or general practice setting.

Interventions in hospital inpatient care, outpatient clinics or home medication reviews will be excluded from this review. In addition, studies that primarily involved a community pharmacist or a community pharmacist providing a service remotely are also excluded. Studies involving paediatric and oncology patients will be excluded as potentially these patients may have a regular follow-up as part of their usual care.

### Search strategy

An initial limited search of MEDLINE, Cochrane Library and CINAHL Plus was undertaken to identify articles on the topic. The text words contained in the titles and abstracts and index terms used to describe the articles of relevant articles were used to develop a full search strategy. The review will aim to include published literature up to March 2022. A systemised search will be conducted using the following electronic databases: MEDLINE, EMBASE, PubMed, Cochrane central register of controlled trials (CENTRAL) Web of Science and NICE Evidence. Reference lists of included full texts will be searched for relevant papers to ensure a comprehensive search of literature. As the review will be looking to inform future research primarily in the United Kingdom, several websites for relevant UK professional organisations will be searched for grey literature relevant to the topic. These include the Royal Pharmaceutical Society, General Pharmaceutical Council, Royal College of General Practitioners, Department of Health, the UK Faculty of Public Health and the National Institute for Health and Care Excellence (NICE) websites. All retrieved literature will be treated in the same manner for eligibility. The search will be limited to English Language. Publications not in English will be excluded due to the cost and time involved in translation. No limitations on the date will be set to ensure an extensive search of literature. Conference abstracts, protocols and case reports will be excluded at this stage due to the limitations of evaluative benefit.

### Search terms

For the scoping review, to gain a comprehensive search of the available literature, the search terms will remain broad in nature. A medical research librarian was consulted to advise on development of the search strategy. The following search terms will be used and will be adapted for each individual database and/or information source, using truncation and Boolean operators. Medical subject headings will be used were appropriate and focused for the research question (Additional file 2).

Two concepts were used for the search strategy and combined with the boolean operator ‘AND’;

*Concept 1:* Pharmacist* adj5 (“primary care” OR clinical OR “practice based” OR “general practi*” OR “GP practice*” OR prescrib* OR “non-dispensing”).

*Concept 2:* “Post hospital discharge” OR “hospital discharge” OR “post hospitalisation” OR “post hospitalization” OR “medic* reconciliation” OR “transfer of care” OR “care transition*” OR “TCAM”.

### Study selection

Based on preliminary findings, this scoping review aims to report findings from qualitative, quantitative, and mixed-method studies. This scoping review will consider both observational and experimental studies including quasi-experimental, randomised controlled trials, non-randomised controlled trials, before and after studies, cohort studies, case–control studies, and cross-sectional studies. Studies published describing the role and impact of primary care pharmacists on the post-hospital discharge care of patients will be included, regardless of the outcome assessed, the profile of patients included or the clinical diagnosis on admission. Identified citations must discuss interventions of pharmacists working in the primary care sector involved in the care of patients who have been recently discharged from hospital.

Following the search, all identified citations will be collated into an online research tool (Rayyan) [43] and duplicates will be removed using the in-built functionality. Titles and abstracts will be screened by two or more independent reviewers for assessment against the inclusion criteria for the review to ensure relevance, then full texts will be examined. A flowchart of the results will be updated throughout the review process to detail search results, duplicates, and screening results. The results of the search will be reported in full in the final scoping review and presented in a PRISMA-ScR flow diagram [41].

Data will be extracted using standardised forms that have been created after a pilot stage and assessed by two or more reviewers. Any disagreements that arise between the reviewers at each stage of the selection process will be resolved through discussion, or with an additional reviewer(s). A pilot stage will be conducted where each member will review a sample of 25 titles/abstracts and then meets to discuss discrepancies. When agreement reaches 75% or greater, the selection of articles can continue [38].

### Data extraction

From the papers included in the scoping review, the following data (Table 1) will be extracted to gather specific details about the participants, concept, context, methods, and key findings related to the review questions. The key elements were selected in accordance with the JBI recommendations for extracting and charting data [38].

The data will be charted in a Microsoft Word table format, to allow analysis and identification of key themes. A draft extraction form will be piloted and modified and as necessary during the process. Any modifications will be detailed in the review. Any disagreements between the reviewers will be resolved through discussion, and/or with additional reviewers. If there is any missing data required, the authors if the papers will be contacted to request this. For charting of process-orientated data, a qualitative content analysis approach will be used [37]. An inductive content analysis approach will most likely be useful in to map key concepts for this review [44].

### Risk of bias or quality assessment

Scoping reviews traditionally do not seek to undertake a risk of bias assessment or assess the quality of evidence [39].

### Data synthesis

The data analysis will take a descriptive numeral analysis approach to examine the extent, nature and distribution of papers included in the review. The extracted data from the charting process will be presented in tabular form mapping the location of studies, types of studies/methodological design and interventions studied. Furthermore, a summarised narrative of the key concepts relating to the review question will be presented to identify any gaps in the literature and inform future research. As scoping reviews typically intend to map the available evidence, the analysis will be quite descriptive unlike systematic reviews which aim to provide a more detailed synthesis [45]. Therefore, the process is anticipated to be iterative, and the analysis will be reviewed throughout and discussed between the reviewers. Recognising the need to strengthen methodological rigour of this scoping review, the PAGER (Patterns, Advances, Gaps, Evidence for practice and Research recommendations) framework [46] will be incorporated to enhance consistency in charting and synthesis of findings. The iterative and flexible use of the PAGER framework would make it a useful tool to meet the needs of this scoping review and will be considered in relation to the research question. This method will aim to classify and summarise the type of evidence available in this field, identifying further research recommendations.

## Discussion

This scoping review will allow the analysis of a broad scope of literature to identify what research has already been conducted in this area. This will also identify gaps in research and inform and guide future research. This scoping review will guide the validity of conducting a future systematic review with an aim to examine methods to improve the transitions of care for patients and collaboration of primary care pharmacists with other health professionals.

It was identified that a scoping review would be the most appropriate methodology for the research question proposed to scope current literature. To our knowledge, little has been researched on the role of primary care pharmacists in transitions of care and this review will allow mapping of the current topic and guide valuable future research. Conversely, systematic reviews would be focused on a particular research question and would restrict the type of studies included in the review hence not providing the required overview in this field. A traditional methodological quality assessment will not be performed as the aim is to understand what has been performed rather than assess the evidence of effectiveness.

The limitations of scoping reviews have been historically criticised for the lack of methodological rigorous quality appraisal of the studies included and the absence of risk of bias assessment. This may impact on the validity of recommendations for future practice. However, this will be minimised by using the most current up-to-date scoping review methodology approach [38] using the PRISMA-ScR guideline [41] and consideration to the quality of evidence using the PAGER framework tool [46], where appropriate.

## Conclusion

In summary, this scoping review will provide an overview of the current evidence of the role and impact of primary care pharmacists in patients recently discharged from hospital. This scoping review will aim to identify any gaps in current research and support implications for future research, policy, and practice.

**Table 1:** Elements for data extraction

Population	Concept	Context	Key findings
Characteristics of participant population Primary care pharmacist intervention (role/setting)	Aim of study Intervention and/or comparator (if applicable) Research methodology	Location/country of study Type of Study design/evidence	Outcome measures reported Financial Impacts (if reported) Collaboration with other health care professionals reported Any gaps in the research identified?

## Supplementary Information

**Additional file 1.** JBI 2020 Approach for scoping reviews—9 steps [38].

**Additional file 2.** Search strategy.

## Data Availability

Not applicable.

## Abbreviations

GP: General practitioner/practice
NHSE: NHS England
NICE: National Institute for Health and Care Excellence
NMAHPs: Non-Medical Allied Health Professionals
MRC: Medical Research Council
PCP: Primary care pharmacists
RCP: Royal College of Physicians
RPS: Royal Pharmaceutical Society
WHO: World Health Organization

## References

[1] Flatman J (2021). How to improve medication safety at hospital discharge: let’s get practical. Future Healthc J..

[2] Alqenae FA, Steinke D, Keers RN (2020). Prevalence and nature of medication errors and medication-related harm following discharge from hospital to community settings: a systematic review key points. Drug Saf.

[3] Viktil KK, Salvesen Blix H, Eek AK, Davies MN, Moger TA, Reikvam A (2012). How are drug regimen changes during hospitalisation handled after discharge: a cohort study. BMJ Open.

[4] Angley M, Ponniah AP, Spurling LK, Sheridan L, Colley D, Nooney VB (2011). Feasibility and timeliness of alternatives to post-discharge home medicines reviews for high-risk patients. J Pharm Pract Res.

[5] Elliott RA, Ann Elliott R, Camacho E, Jankovic D (2021). Economic analysis of the prevalence and clinical and economic burden of medication error in England. BMJ Qual Saf.

[6] Parekh N, Ali K, Stevenson JM, Davies JG, Schiff R, van der Cammen T (2018). Incidence and cost of medication harm in older adults following hospital discharge: a multicentre prospective study in the UK. Br J Clin Pharmacol.

[7] Medication safety in transitions of care [internet] Who.int. 2022. World Health Organization (WHO). 2019. www.who.int/publications/i/item/WHO-UHC-SDS-2019.9. Accessed 27 Dec 2021.

[8] Gao L, Maidment I, Matthews FE, Robinson L, Brayne C (2018). Medication usage change in older people (65+) in England over 20 years: findings from CFAS I and CFAS II. Age Ageing.

[9] Bagge M, Norris P, Heydon S, Tordoff J (2014). Older people’s experiences of medicine changes on leaving hospital. Res Soc Adm Pharm.

[10] Fylan B, Armitage G, Naylor D, Blenkinsopp A (2018). A qualitative study of patient involvement in medicines management after hospital discharge: an under-recognised source of systems resilience. BMJ Qual Saf.

[11] Greysen SR, Hoi-Cheung D, Garcia V, Kessell E, Sarkar U, Goldman L (2014). “Missing pieces”—functional, social, and environmental barriers to recovery for vulnerable older adults transitioning from hospital to home. J Am Geriatr Soc.

[12] Morris RL, Stocks SJ, Alam R, Taylor S, Rolfe C, Glover SW (2018). Identifying primary care patient safety research priorities in the UK: a James Lind Alliance Priority Setting Partnership. BMJ Open.

[13] Picton C. Keeping patients safe when they transfer between care providers—getting the medicines right. Good practice guidance for healthcare professions. London: Royal Pharmaceutical Society, 2011. https://www.aomrc.org.uk/wp-content/uploads/2016/05/Keeping_patients_safe_professional_guidance_0711.pdf. Accessed 22 Oct 2022.

[14] NHS England. NHS Discharge Medicines Service. 2022. https://www.england.nhs.uk/primary-care/pharmacy/nhs-discharge-medicines-service/. Accessed 24 Feb 2022.

[15] National Prescribing Centre. NPC Archive Item: Medicines Management: When patients transfer between care providers – Centre for Medicines Optimisation. 2011 [cited 2021 Dec 28]. Available from: <https://www.centreformedicinesoptimisation.co.uk/medicines-management-when-patients-transfer-between-care-providers/>

[16] RCP Quality Improvement and patient Safety. Medication safety at hospital discharge: improvement guide and resource. London; 2021. https://www.rcplondon.ac.uk/projects/outputs/medication-safety-hospital-discharge-improvement-guide-and-resource. Accessed 27 Dec 2021.

[17] Avery AJ (2017). Pharmacists working in general practice: can they help tackle the current workload crisis?. Br J Gen Pract.

[18] NHS. Five year forward view. 2014. https://www.england.nhs.uk/wp-content/uploads/2014/10/5yfv-web.pdf. Accessed 14 Jan 2022.

[19] Bradley F, Seston E, Mannall C, Cutts C (2018). Evolution of the general practice pharmacist’s role in England: a longitudinal study. Br J Gen Pract.

[20] Alshehri AA, Cheema E, Yahyouche A, Haque·MS, Jalal Z. Evaluating the role and integration of general practice pharmacists in England: a cross-sectional study. Int J Clin Pharm. 2021;43(3):1609–18. 10.1007/s11096-021-01291-6 10.1007/s11096-021-01291-6 PMC8642258 34080088

[21] Ho PM, Lambert-Kerzner A, Carey EP, Fahdi IE, Bryson CL, Melnyk SD (2014). Multifaceted intervention to improve medication adherence and secondary prevention measures after acute coronary syndrome hospital discharge a randomized clinical trial. JAMA Intern Med.

[22] National Institute for Health and Care Excellence (NICE). Medicines Optimisation: the safe and effective use of medicines to enable the best possible outcomes [NG5]. 2015. https://www.nice.org.uk/guidance/ng5. Accessed 14 Jan 2022. 26180890

[23] Foot H, Scott I, Sturman N, Whitty JA, Rixon K, Connelly L (2021). Impact of pharmacist and physician collaborations in primary care on reducing readmission to hospital: a systematic review and meta-analysis. Res Social Adm Pharm.

[24] Redmond et al. Cochrane Library Cochrane Database of Systematic Reviews Impact of medication reconciliation for improving transitions of care (Review). 2018; www.cochranelibrary.com. 10.1002/14651858.CD010791.pub2 PMC6513651 30136718

[25] Mcnab D, Bowie P, Macwalter G, Ryan M, Morrison J (2018). Systematic review and meta-analysis of the effectiveness of pharmacist-led medication reconciliation in the community after hospital discharge. BMJ Qual Saf.

[26] Mekonnen AB, McLachlan AJ, Brien JE (2016). Pharmacy-led medication reconciliation programmes at hospital transitions: a systematic review and meta-analysis. J Clin Pharm Ther.

[27] Holland R, Desborough J, Goodyer L, Hall S, Wright D, Loke YK (2008). Does pharmacist-led medication review help to reduce hospital admissions and deaths in older people? A systematic review and meta-analysis. Br J Clin Pharmacol.

[28] 12.2.1 The GRADE approach Handbook-5-1.cochrane.org. 2022. https://handbook-5-1.cochrane.org/chapter_12/12_2_1_the_grade_approach.htm. Accessed 13 Feb 2022.

[29] Nazar H, Nazar Z, Portlock J, Todd A, Slight SP, Hamde D, et al. A systematic review of the role of community pharmacies in improving the transition from secondary to primary care. Br J Clin Pharmacol. 2015;80(5):936–48. 10.1111/bcp.12718. 10.1111/bcp.12718 PMC4631167 26149372

[30] Medical Research Council (MRC). Developing and evaluating complex interventions: new guidance. 2006. http://www.mrc.ac.uk/documents/pdf/complex-interventions-guidance/.

[31] Foot H, Scott I, Sturman N, Whitty JA, Rixon K, Connelly L (2022). Impact of pharmacist and physician collaborations in primary care on reducing readmission to hospital: a systematic review and meta-analysis. Res Soc Adm Pharm.

[32] Hazen et al. The degree of integration of non-dispensing pharmacists in primary care practice and the impact on health outcomes: A systematic review | Elsevier Enhanced Reader. Res Soc Adm Pharm. 2018;14(3):228–40. https://reader.elsevier.com/reader/sd/pii/S1551741116305794?token=EB4AB0BE48FDB35685628FB05795734BA90C4D3E440D71B2795B33645B0D04B1629136C530A52999F4CE050A756A5F25&originRegion=eu-west-1&originCreation=20220106221344. Accessed 6 Jan 2022. 10.1016/j.sapharm.2017.04.014 28506574

[33] Tan ECK, Stewart K, Elliott RA, George J (2014). Pharmacist services provided in general practice clinics: a systematic review and meta-analysis. Res Soc Adm Pharm.

[34] Ensing HT, Stuijt CCM, van den Bemt BJF, van Dooren AA, Fatma K-Ç, Koster ES, et al. Identifying the optimal role for pharmacists in care transitions: a systematic review. J Managed Care Special Pharm JMCP. 2015;21. Available from: www.amcp.org. 10.18553/jmcp.2015.21.8.614 PMC10397897 26233535

[35] Hayhoe B, Foley K, Majeed A, Ruzangi J, Greenfield G, Acuyo CJ (2019). Impact of integrating pharmacists into primary care teams on health systems indicators: a systematic review. Br J Gen Pract.

[36] Munn Z, Peters MDJ, Stern C, Tufanaru C, McArthur A, Aromataris E (2018). Systematic review or scoping review? Guidance for authors when choosing between a systematic or scoping review approach. BMC Med Res Methodol.

[37] Levac D, Colquhoun H, O’Brien KK (2010). Scoping studies: advancing the methodology. Implement Sci.

[38] Peters M, Godfrey C, McInerney P, Munn Z, Trico A, Khalil H. Chapter 11: scoping reviews. In: JBI Manual for evidence synthesis. JBI; 2020.

[39] Arksey H, O’Malley L (2005). Scoping studies: towards a methodological framework. Int J Soc Res Methodol Theory Pract.

[40] Peters MDJ, Godfrey CM, Khalil H, McInerney P, Parker D, Soares CB (2015). Guidance for conducting systematic scoping reviews. Int J Evid Based Healthc.

[41] Tricco AC, Lillie E, Zarin W, O’Brien KK, Colquhoun H, Levac D (2018). PRISMA extension for scoping reviews (PRISMA-ScR): checklist and explanation. Ann Intern Med.

[42] Gradinger FM, Britten NB, Wyatt K, Froggatt K, Gibson AM, Jacoby AB (2013). Values associated with public involvement in health and social care research: a narrative review. Health Expect.

[43] Ouzzani M, Hammady H, Fedorowicz Z, Elmagarmid A (2016). Rayyan—a web and mobile app for systematic reviews. Syst Rev.

[44] Elo S, Kyngäs H (2008). The qualitative content analysis process. J Adv Nurs.

[45] Pollock D, Davies EL, Peters MDJ, Tricco AC, Alexander L, McInerney P (2021). Undertaking a scoping review: a practical guide for nursing and midwifery students, clinicians, researchers, and academics. J Adv Nurs.

[46] Bradbury-Jones C, Aveyard H, Herber O, Isham L, Taylor J, O’Malley L (2021). Scoping reviews: the PAGER framework for improving the quality of reporting. Int J Soc Res Methodol.

