# Supplementary material for: Role of primary care pharmacists in the post-hospital discharge care of patients: a scoping review protocol
Source: J Pharm Policy Pract. 2022 Oct 29;15:75. doi: 10.1186/s40545-022-00473-5 (PMC9617399; doi:10.1186/s40545-022-00473-5)
Supplement: Supplementary file 1 — Additional file 1. JBI 2020 Approach for scoping reviews—9 steps [38]. [file 40545_2022_473_MOESM1_ESM.docx]

**JBI 2020 Approach for scoping reviews - 9 steps** (Peters et al., 2020)

***Enhancements proposed by Peters et al (2015, 2017, 2020).**

1. Defining and aligning the objective/s and question/s
2. Developing and aligning the inclusion criteria with the objective/s and question/s
3. Describing the planned approach to evidence searching, selection, data extraction, and presentation of the evidence.
4. Searching for the evidence
5. Selecting the evidence
6. Extracting the evidence
7. Analysis of the evidence
8. Presentation of the results
9. Summarizing the evidence in relation to the purpose of the review, making conclusions and noting any implications of the findings
