# Supplementary material for: Role of primary care pharmacists in the post-hospital discharge care of patients: a scoping review protocol
Source: J Pharm Policy Pract. 2022 Oct 29;15:75. doi: 10.1186/s40545-022-00473-5 (PMC9617399; doi:10.1186/s40545-022-00473-5)
Supplement: Supplementary file 2 — Additional file 2. Search strategy. [file 40545_2022_473_MOESM2_ESM.docx]

| **Database** | **Search Terms** | **Date Search Run** | **Number of results** | **Number of eligible papers** |
| --- | --- | --- | --- | --- |
| **Ovid MEDLINE(R) and In-Process, In-Data-Review & Other Non-Indexed Citations <1946 to March 17, 2022>** | 1 (Pharmacist* adj5 ("primary care" or clinical or "practice based" or "general practi*" or "GP practice*" or prescrib* or "non-dispensing")).mp. [mp=title, abstract, original title, name of substance word, subject heading word, floating sub-heading word, keyword heading word, organism supplementary concept word, protocol supplementary concept word, rare disease supplementary concept word, unique identifier, synonyms] 7420  2 Patient Discharge/ or "post-hospital discharge".mp. 35832  3 Patient Discharge/ or "post-hospitalisation".mp. or Hospitalization/ 157179  4 Hospitalization/ or Patient Discharge/ or "post hospitalisation".mp. 157179  5 Hospitalization/ or Patient Discharge/ or "post-hospitalization".mp. 157428  6 Hospitalization/ or Medication Reconciliation/ or "medic* reconciliation".mp. 127984  7 Patient Transfer/ or "transfer of care".mp. 9853  8 Patient Discharge/ or "Continuity of Patient Care"/ or Patient Transfer/ or Transitional Care/ or "care transition*".mp. 63266  9 "TCAM".mp. 177  10 2 or 3 or 4 or 5 or 6 or 7 or 8 or 9 186087  11 1 and 10 693  12 limit 11 to english language 672 |  |  |  |
| Embase <1974 to 2022 March 16> | 1 (Pharmacist* adj5 ("primary care" or clinical or "practice based" or "general practi*" or "GP practice*" or prescrib* or "non-dispensing")).mp. [mp=title, abstract, heading word, drug trade name, original title, device manufacturer, drug manufacturer, device trade name, keyword heading word, floating subheading word, candidate term word] 16858  2 *hospital discharge/ or "post hospital discharge".mp. 15539  3 "post hospitalisation".mp. or *hospital discharge/ 14985  4 *hospital discharge/ or "post hospitalization".mp. 15928  5 *medication reconciliation/ or "medic* reconciliation".mp. 7471  6 "transfer of care".mp. 1214  7 "care transition*".mp. 3520  8 "TCAM".mp. 258  9 2 or 3 or 4 or 5 or 6 or 7 or 8 28159  10 1 and 9 1390  11 limit 10 to english language 1337 |  |  |  |
| PubMed | ("pharmacist*"[Title/Abstract] AND ("primary care"[Title/Abstract] OR "clinical"[Title/Abstract] OR "practice based"[Title/Abstract] OR "general practi*"[Title/Abstract] OR "gp practice*"[Title/Abstract] OR "prescrib*"[Title/Abstract] OR "non-dispensing"[Title/Abstract]) AND "english"[Language] AND (("Post hospital discharge"[Title/Abstract] OR "post hospitalisation"[Title/Abstract] OR "post hospitalization"[Title/Abstract] OR "medicines reconciliation"[Title/Abstract] OR "transfer of care"[Title/Abstract] OR "care transition*"[Title/Abstract] OR "TCAM"[Title/Abstract]) AND "english"[Language])) AND (english[Filter])  As PubMed was failing to recognise N5 and “medic* reconciliation” |  |  |  |
| Cochrane central register of controlled trials (CENTRAL) | #1 Pharmacist* adj5 ("primary care" or clinical or "practice based" or "general practi*" or "GP practice*" or prescrib* or "non-dispensing") 135  #2 "post hospital discharge" 232  #3 "post hospitalisation" 218  #4 "post hospitalization" 218  #5 "medic* reconciliation" 0  #6 "transfer of care" 52  #7 "TCAM" 8  #8 #2 OR #3 OR #4 OR #5 OR #6 OR #7 501  #9 #1 AND #8 6 |  |  |  |
| Web of Science | #1 AK=(Pharmacist* AND("primary care" or clinical or "practice based" or "general practi*" or "GP practice*" or prescrib* or "non-dispensing")) = 1864  #2 AK=("Post hospital discharge" OR "post hospitalisation" OR "post hospitalization" OR "medic* reconciliation" OR "transfer of care" OR "care transition*" OR "TCAM")  =1660  #1 AND #2 = 40  Limit - English Language = 38 |  |  |  |
| NICE Evidence. | (Pharmacist* AND("primary care" OR clinical OR "practice based" OR "general practi*" OR "GP practice*" OR prescrib* OR "non dispensing")) AND ("Post hospital discharge" OR "post hospitalisation" OR "post hospitalization" OR "medic* reconciliation" OR "transfer of care" OR "care transition*" OR "TCAM") |  |  |  |

| **Websites of Relevant Professional Organisations searched** | **Search Terms** | **Date Search Run** | **Number of results** | **Number of eligible papers** |
| --- | --- | --- | --- | --- |
| **Royal Pharmaceutical Society** | (Pharmacist* AND("primary care" OR clinical OR "practice based" OR "general practi*" OR "GP practice*" OR prescrib* OR "non dispensing")) AND ("Post hospital discharge" OR "post hospitalisation" OR "post hospitalization" OR "medic* reconciliation" OR "transfer of care" OR "care transition*" OR "TCAM")  (Pharmacist* N5 "primary care" OR clinical OR "practice based" OR "general practi*" OR "GP practice*" OR prescrib* OR "non dispensing")) AND ("Post hospital discharge" OR "post hospitalisation" OR "post hospitalization" OR "medic* reconciliation" OR "transfer of care" OR "care transition*" OR "TCAM")    (Pharmacist* AND ("Post hospital discharge" OR "post hospitalisation" OR "post hospitalization" OR "medic* reconciliation" OR "transfer of care" OR "care transition*" OR "TCAM")  Pharmacist AND "post-hospital discharge" |  |  |  |
| **General Pharmaceutical Council** | Primary care pharmacist AND post hospital discharge  OR (pharmacist AND transfer of care) |  |  |  |
| **Royal College of General Practitioners** | Primary care pharmacist AND post hospital discharge  Pharmacist* AND post hospital discharge  Pharmacist AND post hospital discharge  pharmacist AND transfer of care |  |  |  |
| **Department of Health** | (Pharmacist* N5 "primary care" OR clinical OR "practice based" OR "general practi*" OR "GP practice*" OR prescrib* OR "non dispensing")) AND ("Post hospital discharge" OR "post hospitalisation" OR "post hospitalization" OR "medic* reconciliation" OR "transfer of care" OR "care transition*" OR "TCAM")) |  |  |  |
| **The UK faculty of public health website and the Journal of Public Health** | (Pharmacist* AND("primary care" OR clinical OR "practice based" OR "general practi*" OR "GP practice*" OR prescrib* OR "non dispensing")) AND ("Post hospital discharge" OR "post hospitalisation" OR "post hospitalization" OR "medic* reconciliation" OR "transfer of care" OR "care transition*" OR "TCAM")  Pharmacist AND post hospital discharge  Pharmacist AND transfer of care |  |  |  |
| **National Institute for Health and Care Excellence (NICE)** | (Pharmacist* AND("primary care" OR clinical OR "practice based" OR "general practi*" OR "GP practice*" OR prescrib* OR "non dispensing")) AND ("Post hospital discharge" OR "post hospitalisation" OR "post hospitalization" OR "medic* reconciliation" OR "transfer of care" OR "care transition*" OR "TCAM")  Pharmacist AND post hospital discharge  Pharmacist AND transfer of care |  |  |  |
